# Imaging diagnosis of aspergilloma

**DOI:** 10.3402/jchimp.v2i1.17276

**Published:** 2012-04-30

**Authors:** Chantal Youssef, David M. Widlus

**Affiliations:** 1 Department of Medicine, Union Memorial Hospital, Baltimore, Maryland, USA; 2 Department of Radiology, Union Memorial Hospital, University of Maryland School of Medicine, Baltimore, Maryland, USA

A 55-year-old man with Human Immunodeficiency Virus (HIV) infection and Acquired Immunodeficiency Syndrome (AIDS), has a history of non-compliance to antiretroviral therapy with recurrent opportunistic infections due to clostridium difficile and mycobacterium avium complex. He presented to the hospital with fever, diarrhea, weight loss and generalized weakness, and was diagnosed with clostridium difficile colitis and treated as such. His absolute CD4 count was 153 cells/microliter and HIV1-RNA 2,190 copies/mL.

**Fig. 1:**
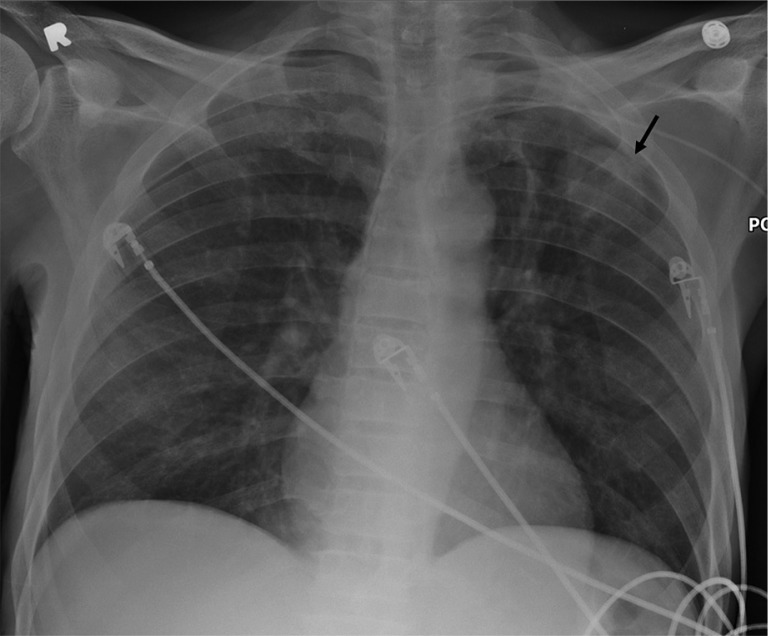
Left upper lobe cavitary area is seen with intra-cavitary mass (arrow). PICC line is incidentally noted.

**Fig. 2:**
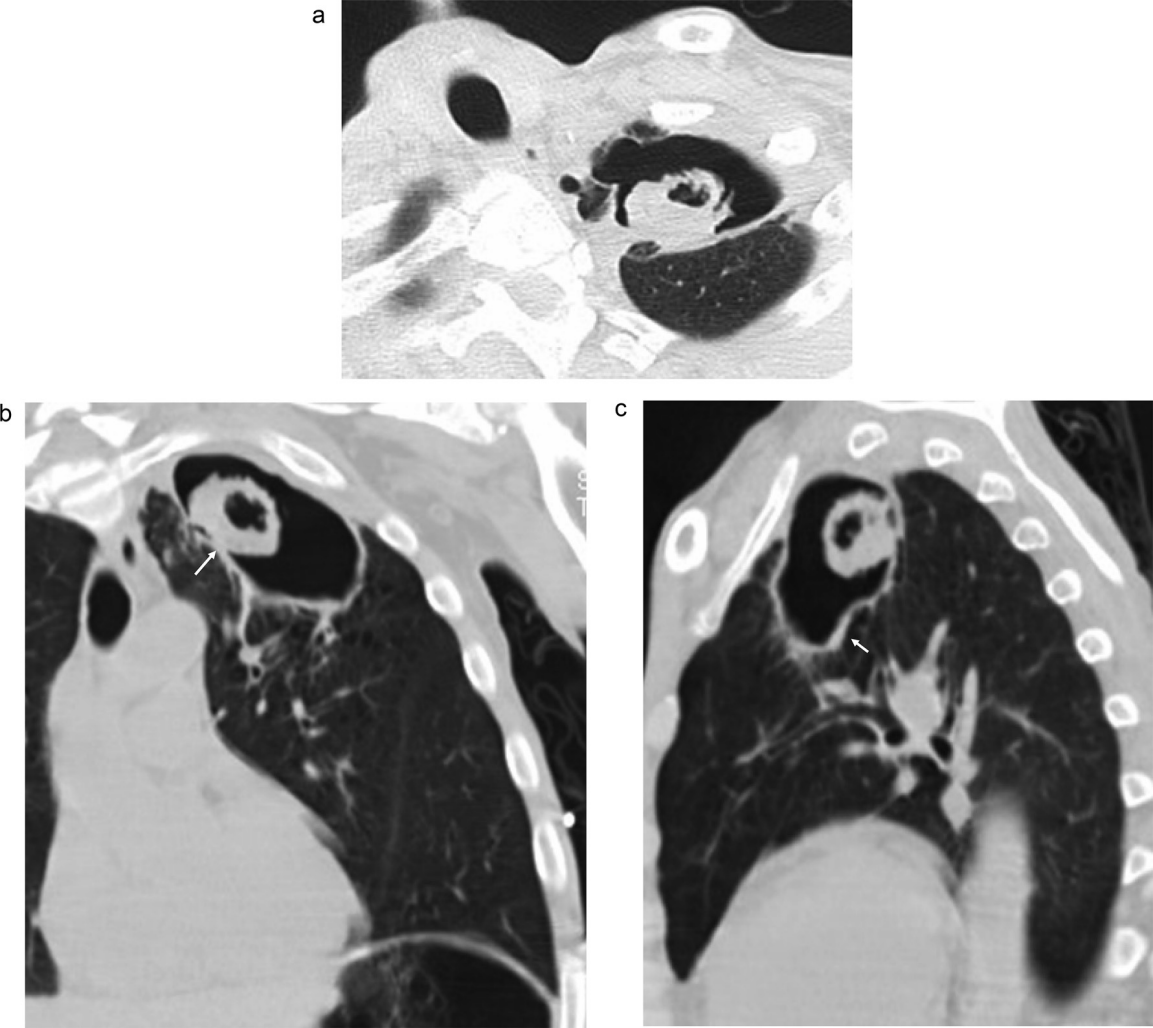
(a,b,c) Axial, coronal and sagittal CT scans of the chest show the intra-cavitary mass with surrounding ‘air-crescent’ (Fig. 2b arrow). The wall of the cavity is thickened (Fig. 2c arrow).

Chest radiograph showed a left upper lobe lung cavity containing a soft tissue component of 4 cm compatible with mycetoma (Fig. 1). This finding was subsequently confirmed by a chest CT scan (Figs. 2a,b,c).

Aspergillus is a common saprophyte living in the soil and plants, transmitted to humans by inhalation. Tissue invasion is uncommon and occurs most frequently in the setting of immunosuppression. In HIV-infected patients, aspergillosis is mostly seen in untreated patients with advanced AIDS. One of the risk factors for aspergillosis in HIV-infected patients is the presence of underlying lung disease such as a prior pneumocystis jirovecci pneumonia or cavitary disease.

The patient can have one of four different clinical presentations:

1. Invasive pulmonary aspergillosis with fever, cough, dyspnea, pleuritic chest pain, anorexia and weight loss.
2. Tracheobronchial disease which can lead to extensive inflammation and invasion of the tracheobronchial tree.
3. Aspergilloma is a non-invasive form of colonization by Aspergillus in a prior lung cavity, which is usually asymptomatic but can present with fever, chest pain, cough, hemoptysis, fatigue and weight loss. The etiology is the contagious spore form of the organism.
4. Allergic bronchopulmonary aspergillosis, very rare entity among HIV-infected patients.

This patient had the asymptomatic presentation of aspergilloma which arose in a pre-existing lung cavity, most likely secondary to his prior mycobacterium avium complex infection. In this form the fungal spores are typically inhaled into an established lung cavity where they are able to multiply. Eventually a fungus ball composed of aspergillus hyphae, inflammatory cells, fibrin, mucus and cellular debris forms.

Diagnosis of aspergilloma is made by typical chest radiographic and CT findings, in the correct clinical setting, combined with sputum cultures or serum antibodies to aspergillus.

The radiographic findings typical for aspergilloma include:

1. Cavitary lesion, most commonly in the upper lobes. Wall thickening is a sign of secondary infection such as with an aspergilloma.
2. Intra-cavitary mass, with an air crescent sign, often easier to appreciate on CT scan than on Chest radiograph. The mass can often be seen to move within the cavity with change in patient position.

Treatment recommendations vary. Many patients with a single aspergilloma, who are asymptomatic and have stable radiographic findings over many months, require only continued observation. Embolization or surgical resection are usually offered to prevent or treat hemoptysis.
